# Seasonal Variations in Macrobenthos Communities and Their Relationship with Environmental Factors in the Alpine Yuqu River

**DOI:** 10.3390/biology14020120

**Published:** 2025-01-24

**Authors:** Jianmin Ge, Jianyong Chen, Fangze Zi, Tianjian Song, Linghui Hu, Zhouminkang He, Lei Wu, Yandong Ding, Hongtao Li

**Affiliations:** 1 College of Animal Science and Technology, Tarim University, Alar 843300, China; 17788615648@163.com (J.G.); 10757222068@stumail.taru.edu.cn (L.H.); 2 Wuhan Zhongke Ruihua Ecological Technology Co., Ltd., Wuhan 430063, China; chenjianyong@sinoeco.net (J.C.); dingyandong@sinoeco.net (Y.D.); 3 College of Material Science and Engineering, Beijing University of Chemical Technology, Beijing 100029, China; 4 College of Water Sciences, Beijing Normal University, Beijing 100875, China; 202131470020@mail.bnu.edu.cn; 5 State Key Laboratory of Environmental Criteria and Risk Assessment, Chinese Research Academy of Environmental Sciences, Beijing 100012, China; 6 Zhenxi Town People ’s Government of Weiyuan County, Neijiang 641000, China; 706994822@163.com; 7 College of Fisheries, Huazhong Agricultural University, Wuhan 430070, China; wulei@sinoeco.net

**Keywords:** macrobenthos, seasonal variation, plateau river, South Asia

## Abstract

This study investigated the spatial and temporal variations of macrobenthos community structure in the Yuqu River Basin during the dry and wet seasons due to environmental factors. This study quantified the independent and interactive contributions of hydrophysical, hydrochemical, and climatic factors to the community structure through a variance decomposition analysis (VPA). The study findings indicate that during May (the dry season), factors such as water depth, flow velocity, dissolved oxygen, and air temperature exhibit relatively minor fluctuations, rendering the aquatic environment more stable than in the rainy season. This stability is particularly conducive to the maintenance of the macrobenthic community structure and species diversity, which is especially evident in aquatic insects with nesting habits, such as those belonging to the Trichoptera order (including genera like *Glossosoma*, *Glossosomatidae*, and *Georodes*). In contrast, during August (the rainy season), substantial precipitation alters the thermal conditions of the river, increases flow velocity, raises water levels, and introduces a significant influx of organic matter through sedimentation. This distinctive ecological environment fosters unique adaptive strategies among macrobenthic organisms. Notwithstanding a notable decline in species diversity during this particular phase, there is a concurrent increase in the abundance of individual organisms, which is indicative of the populations’ remarkable capacity to swiftly adapt to environmental heterogeneity. Research has demonstrated that macrobenthic communities within the Yuqu River Basin adopt pronounced adaptive tactics that vary significantly between seasons. During the dry season, these macrobenthic fauna rely heavily on the stability of their physical habitat. In stark contrast, they are compelled to navigate and cope with the more intricate and dynamic changes in hydrological and chemical conditions that characterize the rainy season. The presented results uncover the sensitive responsiveness of the macrobenthic fauna to seasonal hydrological and environmental fluctuations in high-altitude river systems and their adaptive strategies under diverse ecological stressors. Arthropods, in particular, exhibit a marked sensitivity to seasonal hydrological and environmental changes. This study delves into the biodiversity of high-altitude river ecosystems, analyzing the ecological environment and the distribution patterns and seasonal variation characteristics of macrobenthic communities. This study aims to examine how diverse seasons and hydroclimatic conditions modulate the composition of macrobenthic assemblages within the tributaries and principal channels of high-altitude river systems, thereby establishing a foundational reference for future water ecosystem assessments in such regions.

## Simple Summary

This study investigated the changes in macrobenthos fauna (organisms living at the bottom of the river) in the Yuqu River Basin of the Tibet Autonomous Region of China during the dry and rainy seasons and their responses to environmental changes. The environmental changes in the rainy season require these organisms to adapt more to the changing water flow and nutrient conditions than in the dry season. In the dry season, they rely on a stable environment for survival; in the rainy season, they need to adapt quickly to more drastic environmental changes. These findings contribute significantly to a refined understanding of the biodiversity within alpine river ecosystems, and they provide critical insights into the management and conservation strategies for the biota inhabiting these aquatic systems.

## 1. Introduction

In recent years, the issue of global biodiversity conservation has attracted much attention from the international community, including the spatial distribution pattern of biodiversity, which has become the focus of current research in the field of biology [1,2,3,4]. Usually, the damage leading to biodiversity is mainly attributed to climate change [5], habitat destruction [6], environmental pollution [7], and other pernicious drivers [8]. According to related studies, altitude is one of the critical ecological gradients affecting biodiversity [9], and the altitudinal gradient integrates the gradient effects of several environmental factors, such as water temperature, pH, dissolved oxygen microbial activity, etc. With the change in altitude, these ecological parameters undergo regular changes, affecting species distribution and environmental niche differentiation [10,11,12]. Therefore, analyzing the interaction of biodiversity with altitude and environmental factors is crucial for the fields of biogeography and ecology [9,13].

River ecosystems, as representatives of running water ecosystems, bear not only natural functions, such as maintaining species diversity, material circulation, self-purification, and climate regulation, but also have social functions, such as water supply, transport and shipping, power generation, and cultural heritage, and healthy river ecosystems are crucial for human survival and development [14,15,16]. Macrobenthos is located in the middle of the food chain of river ecosystems and is an essential part of the biological community in river ecosystems. It plays the role of connecting primary producers and advanced consumers [17,18]. It promotes the vertical transfer of energy and material in the ecosystem through feeding behavior. It is one of the critical organisms regulating the energy flow and material cycle in river ecosystems. In addition, macrobenthos plays an indispensable role in maintaining the structural and functional stability of river ecosystems by influencing the composition of river microbial communities and the structure of river substrates through their bioturbation effects [19]. Compared with other aquatic animals, macrobenthos is characterized by diverse species, weak migratory ability, wide distribution, a long lifecycle, stable habits, and poor tolerance to environmental stress [20,21]. In addition, they are easy to collect, and the species composition, community structure, seasonal dynamics, and spatial distribution of macrobenthos can effectively reflect the changes in the water environment and aquatic ecological conditions; thus, they are widely used in river environmental monitoring and health assessment [22,23,24,25]. However, at this stage, most studies on macrobenthos community structure focus on functional ecology [19,26,27] and species diversity [20,25,28], and the study areas are primarily concentrated in lakes [22,23], small and medium-sized rivers [29], estuaries [21,30] or coastal regions [25,31]. Conversely, the field of research into the structural composition of macrobenthic communities within high-altitude river systems, as well as their temporal and spatial distributional characteristics, remains relatively underexplored. Only a limited number of findings have emerged from studies led by Figueroa [32], Jacobsen [33], Benjamin [34], and a few others. The dearth of data on large macrobenthic organisms in high-altitude rivers significantly impairs our ability to gain a comprehensive understanding of the functional dynamics of these riverine ecosystems. Consequently, there is an acute need for additional research to be conducted in this area.

The Yuqu River is a first-class tributary on the left bank of the middle reaches of the Nujiang River, originating at the southern foot of the Wahe Mountains near Leiwuqi County in the Changdu area of the Tibet Autonomous Region of China. The basin area of the Yuqu River is about 9379 km^2^, the total length of the mainstream is about 444.3 km, the elevation of the river is 3122 m, and the average flow rate of the estuary is 114 m^3^/s. As a crucial surface water resource for the industrial, agricultural, and residential needs of the people along both banks, the Yuqu River has been increasingly threatened by the proliferation of industrial and agricultural pollutants discharged into its waters, which pose a severe risk to the water quality safety of the river. This contamination also constitutes a potential threat to the composition of the aquatic biodiversity within the river. Therefore, it is of paramount importance and urgency to comprehensively understand the current status and distributional patterns of the aquatic biotic resources in this water body.

This study selected 39 reaches of the main stem and tributaries of the Yuqu River and conducted a longitudinal investigation into the composition and distribution of large macrobenthic macrobenthos, as well as their abundance and temporal variation, during both May (dry season) and August (rainy season) of 2020. This study preliminarily analyzed the trends in the structural changes in the macrobenthic macroinvertebrate communities within the Yuqu River drainage basin and their responses to the seasonal alternation of the high-altitude riverine habitats. The findings aim to provide a theoretical foundation and basic data for the protection of the ecological environment of the Yuqu River system and for ensuring biodiversity during the period of industrial and agricultural development surrounding the river system.

## 2. Materials and Methods

### 2.1. Research Area

The study area includes the entire main stem of the Yuqu River drainage basin, which spans a total length of 443 km and 11 major tributaries, including the Duoqingqu River, Kaichu River, and Dengqu River, which flow through the counties of Leiwuqi, Chaya, Basu, and Zuogong in Changdu City, Tibet Autonomous Region, China. The upper part of the Yuqu River Basin is in a semi-arid and semi-humid area, the upper part of the basin has a plateau temperate semi-humid climate, and the middle and lower reaches of the basin have a plateau temperate semi-arid monsoon climate. The Yuqu River Basin extends over a significant geographical range, with climatic conditions varying across its different regions. The mean annual sunshine duration across the basin areas is between 2100 and 2700 h per year, and the average annual temperature stands at 8 °C. The spatial and temporal distribution of precipitation within the basin is uneven, with the majority of rainfall occurring from June to September. The mean annual precipitation is 484 mm, and the annual evaporation rate ranges from 1032 to 2493 mm, typically exceeding the amount of precipitation. The mismatch between the water and heat conditions creates apparent flood and dry periods in the basin.

**Figure 1:**
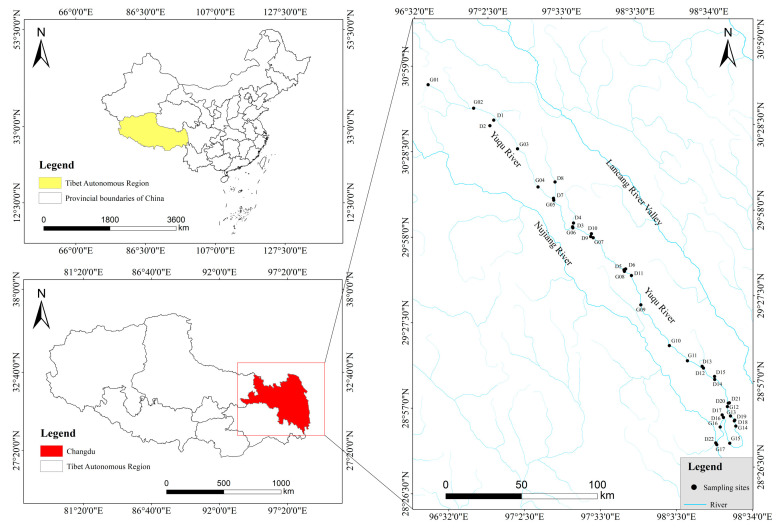
Sample distribution map of the Yuqu River watershed.

In this study, which concerns the “Technical Specification for Freshwater Biological Survey”, “Technical Specification for Ecological Environment Survey and Observation in Nature Reserves”, “Technical Provisions for Survey and Assessment of Inland macrobenthos Invertebrate Diversity”, and other related materials, and incorporates preliminary observations and the spatiotemporal characteristics of the Yuqu River drainage basin, a series of 17 sampling sites were established along the main stem of the river, following a continuous gradient of altitude (from high to low). Additionally, in accordance with the principle of representativeness, 21 sampling sites were set up along 11 larger tributary reaches of the Yuqu River, as well as a single sampling site at the confluence of the Yuqu River and the Nujiang River. All sampling sites were selected under conditions of construction safety and environmental suitability. Detailed information on the sampling sites can be found in Figure 1 and Table S1.

### 2.2. Sample Collection and Processing

In May and August 2020, during the dry and rainy seasons, respectively, two large-scale surveys of macrobenthos were conducted in the Yuqu River Basin. All macrobenthic animal samples collected at each site were processed and stored in accordance with the standards set forth in the “Technical Guidelines for Biodiversity Monitoring—Freshwater macrobenthic Macroinvertebrates” (HJ 710.8—2014). Given the perilous terrain and the rapid flow of the Yuqu River, sampling was conducted using a Peterson grab (RM44-PBS-411 + Beijing Haifuda Technology Co., Ltd. + China + Beijing) and a D-shaped net (DN-512 + Wuhan Peterson Technology Co., Ltd. + China + Wuhan). At each sampling location, observations were conducted along both banks of the river. The selection between the Peterson grab and the D-shaped net was contingent upon the varying hydrological conditions. In situations in which the water depth was considerable and diving was perilous, a 1/16 m^2^ Peterson grab was utilized. At each site, sampling was conducted 8 to 16 times, with the results being pooled together. In areas with less water depth and where safety was ensured, a 30 cm × 30 cm D-shaped net was employed. This net was placed in close proximity to the riverbed and moved upstream against the current for about 1 m to facilitate the collection of samples through the combined action of stirring and water flow. This procedure was repeated three times before the samples were combined. During the cleaning process, all collected mud and sandstone samples were transferred to plastic sampling boxes (Shanghai Chemical Laboratory Equipment Co., Ltd. + China + Shanghai). The initial sediment was first flushed out by washing the samples through a D-shaped net in water. All samples, including those that were not fully cleaned, were then meticulously selected and placed in plastic resealable bags (Shanghai Chemical Laboratory Equipment Co., Ltd. + China + Shanghai). A 75% ethanol solution (Nanjing Chemical Reagent Co., Ltd. + China + Nanjing) was added, and the bag was sealed, labeled with the collection location, sample number, date, and collector, and then taken back to the laboratory for further screening and sorting. In the laboratory, each site’s samples were further washed and sorted. The samples were placed in a 40-mesh sieve (Xinxiang Xinmingde Machinery Co., Ltd. + China + Xinxiang) and the sieve bottom was gently shaken in a basin of clean water to remove any remaining silt. Debris, such as stones, plant twigs, and leaves, were picked out, and the entire sample was then transferred to a white porcelain plate (Shanghai Chemical Laboratory Equipment Co., Ltd. + China + Shanghai) for sorting. All macrobenthic animals in each sample were sorted out and placed into sample bottles (Shanghai Chemical Laboratory Equipment Co., Ltd. + China + Shanghai), to which a 95% ethanol solution (Nanjing Chemical Reagent Co., Ltd. + China + Nanjing) was added for preservation.

All samples were classified, identified, and counted under a microscope or dissecting scope. During the classification and identification of macrobenthic animal samples, we mainly referred to the “Systematic Classification, Biology and Ecology of Freshwater Invertebrates” [35], “Macrobenthos and Ecological Evaluation of Streams” [36], “Journal of the Economic Zoology of China—Freshwater Mollusks” [37], “Fauna of China: Insecta” [38], and other relevant literature on macrobenthos specimens. Among these, some species of Coleoptera, Hemiptera, and Diptera were identified at the family level; groups such as Ephemeroptera, Trichoptera, and Odonata were identified at the genus level, with Ephemeroptera, Plecoptera, and Trichoptera classified under the EPT taxonomic unit. The family Chironomidae is identified to the genus level, while non-Chironomidae aquatic insects are generally identified to the genus or family level. After identifying macrobenthos in each taxonomic unit, they were counted and weighed to calculate each species’ density (ind. m^2^) and biomass (g/m^2^). The specimens were weighed on filter paper to absorb the surface water and then weighed on an electronic balance (FA1004B + Shanghai Precision Instrument Co., Ltd. + China + Shanghai) (accurate to 0.0001 g). The biomass was calculated using a uniform wet weight.

### 2.3. Environmental Factor Measurement

Hydrological and physical variables were measured at each sampling site. Latitude (Lat), longitude (Lon), and altitude (ASL) were measured in situ at each sampling site using a GPS (GM101 + Nanjing Huantian Precision Instrument Co., Ltd. + China + Nanjing); stream width (W) was measured in situ using an infrared laser rangefinder (SW-M100 + Shendawei Technology Co., Ltd. + China + Guangzhou); water depth (WD) and water volume flow rate (WFR) were measured in situ using a portable streamflow meter (FP11 + Global Water + USA + New York, NY); barometric pressure (Pa); secchi disk depth (SDD) was measured in situ using a Seychelles disk (SATO + Shenzhen Fuchi Electronic Technology Co., Ltd. + China + Shenzhen); and water temperature (WT), pondus hydrogenii (pH), dissolved oxygen (DO), conductivity (Cond), total dissolved solids (TDS), and oxidation-reduction potential (ORP) were measured in situ in the water column at each sampling point using a portable water quality analyzer (YSI-556MPS, Beijing Huayitongtai Technology Co., Ltd. + China + Beijing). In addition, 1 L of water samples were collected at each sampling point and transported to the laboratory under refrigeration to determine chemical indicators of water quality, including total nitrogen (TN), total phosphorus (TP), ammonia nitrogen (NH_3_-N), nitrate ion (NO_3_^−^), chemical oxygen demand (COD), etc., which were detected and analyzed in accordance with the Environmental Quality Standard for Surface Water (GB3838-2002) [39].

Potassium persulphate oxidation-ultraviolet spectrometry was used to determine the total nitrogen content of the water samples at each point; ammonium molybdate spectrometry was used to determine the total phosphorus content of the water samples at each end; the ammonia nitrogen content of the water samples was determined using the nano-reagent colorimetric method; the nitrate ion content of the water samples was determined using ion chromatography; and the chemical oxygen demand of the water samples at each point was determined using the potassium dichromate method.

### 2.4. Data Analysis

The primary work of data analysis and graph processing was performed using Excel 2021; the “vegan” and “ggplot2” function packages in R 4.0.4 software were used for the detrended correspondence analysis (DCA) to analyze the relationship between macrobenthos communities and environmental factors in the Yuqu River. The canonical correlation analysis (CCA) or redundancy analysis (RDA) was selected according to the results of the gradient ranking axis.

To assess the relative contribution of different environmental factors to the macrobenthos community structure in the Yuqu River Basin, this study adopted the variance decomposition analysis (VPA) method, which was performed by using the “vegan” package in R. Specifically, the carport() function was used to decompose the relative effects of multiple environmental factors. The decomposition was performed using the varpart() function. In the analysis, the macrobenthos community data matrix was used as the response variable, and the hydrophysical, hydrochemical, and climatic factors were imported into the carport() function as the explanatory variables to decompose the variance. For example, where response_data denotes the species composition data matrix of the macrobenthos community, and explanatory_data1, explanatory_data2, and explanatory_data3 denote the data matrices of physical, chemical, and climatic factors (temperature and precipitation), respectively. The VPA analysis enables the quantification of the independent contributions of each type of environmental factor to the variation in macrobenthos community structure, as well as the joint effects of the interactions between the factors on the community structure, to better understand the roles of different environmental factors in seasonal variation and their relative importance. This analytical approach reveals the different driving roles of hydrophysical, chemical, and climatic factors on macrobenthos community structure in the dry and wet seasons and, in particular, how to cope with the effects of seasonal variation by adjusting environmental adaptation strategies.

## 3. Results

### 3.1. Spatiotemporal Distribution Characteristics of Macrobenthos Communities

**Figure 2:**
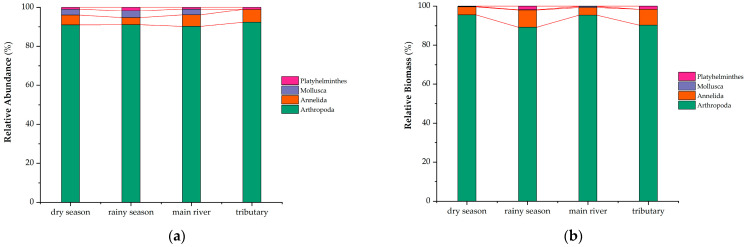
Composition of species and individual numbers of macrobenthos in the Yuqu River Basin across different seasons: (**a**) species quantity composition of macrobenthos; (**b**) individual quantity composition of macrobenthos.

As illustrated in Table 1 and Table S2 and Figure 2, a total of 3319 macrobenthic animal samples were collected in this study. Upon identification, these samples comprised four phyla, 89 genera, and 102 species. Arthropoda exhibited the highest relative species abundance, accounting for 91%, 91%, 90%, and 92% of the samples collected during the dry season, rainy season, mainstream, and tributaries, respectively. Annelida followed, with proportions of 5%, 4%, 6%, and 7% in the dry season, rainy season, mainstream, and tributaries, respectively. Moreover, Arthropoda also showed a significant contribution to the relative biomass, representing 96%, 89%, 95%, and 90% of the biomass in the dry season, rainy season, mainstream, and tributaries, respectively. In contrast to the rainy season, the Yuqu River Basin exhibited a higher macrobenthic species diversity during the dry season, with a total of 100 species documented. Despite this, the biomass of macrobenthic organisms in the dry season (1490 individuals) was found to be lower than that in the rainy season (1829 individuals). Significant disparities were noted in both species richness and biomass between the two seasons. Geographically, there was a minimal variation in the number of macrobenthic species between the mainstream (102 species) and the tributaries (91 species). However, the biomass of macrobenthic organisms in the tributaries (2166 individuals) was notably greater than in the mainstream (1153 individuals). The Platyhelminthes exhibited a relatively low representation in the current survey, with only a single species identified. Furthermore, during the present survey, only a single nematode individual was encountered, an occurrence that likely reflects considerable randomness. Consequently, this finding was excluded from the computational dataset.

**Figure 3:**
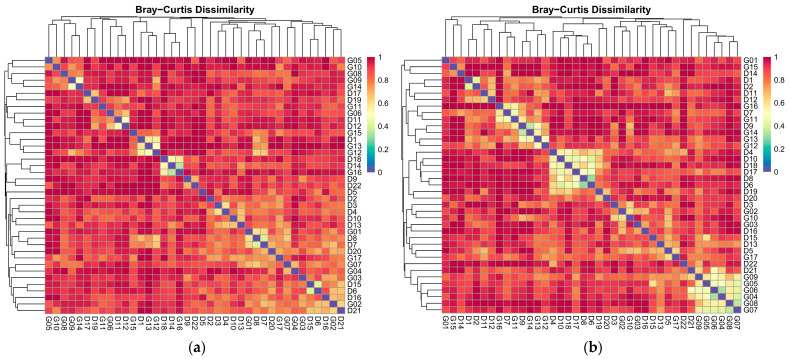
Bray–Crutis cluster heat map of macrobenthos invertebrate communities in different seasons in Yuqu River Basin: (**a**) dry season; (**b**) rainy season. Figures labeled with “G” denote sampling sites located in the mainstream section of the Yuqu River, while those labeled with “D” indicate sites situated in the tributary sections of the Yuqu River.

Heatmaps based on the Bray–Curtis dissimilarity index show the similarity and dissimilarity between the different sampling sites. The relationship between the samples in the dry season is reflected by the color shades (Figure 3a), with lighter colors representing higher similarity and darker colors indicating higher dissimilarity. A high degree of similarity is shown between G05 and G10, suggesting that they are closer regarding community structure. G08 and D9 are significantly different from each other, indicating a considerable difference in species abundance and community structure composition between the two sampling sites. Similarly, D7 and D5 were less different and might be in similar ecosystems, while G16 and D17 showed significant differences, suggesting that they were from different ecosystems or influenced by different environmental factors. In addition, G07 and G17 showed a high degree of similarity, indicating that they share more community characteristics, while G01 and G02 were more different and may be in different ecological conditions. Overall, G05 and G10, G07, and G17 showed high similarity among samples, while G08 and D9 and G16 and D17 revealed significant differences in community composition, reflecting the diversity of ecosystems or environmental conditions.

In the heatmap of the Bray–Curtis dissimilarity index in the rainy season (Figure 3b), there is a high similarity between G01 and G15, which may be located in similar ecological environments. While G09 and G05 show significant dissimilarity, they differ in biotope structure or environmental conditions. D18 and D19 show very high similarity, and their species compositions and ecological conditions are very close. The community compositions between D9 and D10 are similar and may be influenced by the same environmental factors. However, G02 and G10 show a high degree of dissimilarity, possibly reflecting that they are in different environmental conditions or community characteristics. Overall, G01 and G15, as well as D18 and D19, show high similarity, indicating that their ecological environments and community structures are similar. At the same time, G09 and G05, as well as G08 and G07, reflect significant environmental differences between them, revealing the diversity of biological communities in different environments.

### 3.2. Impact of Environmental Factors on Macrobenthos Communities

**Figure 4:**
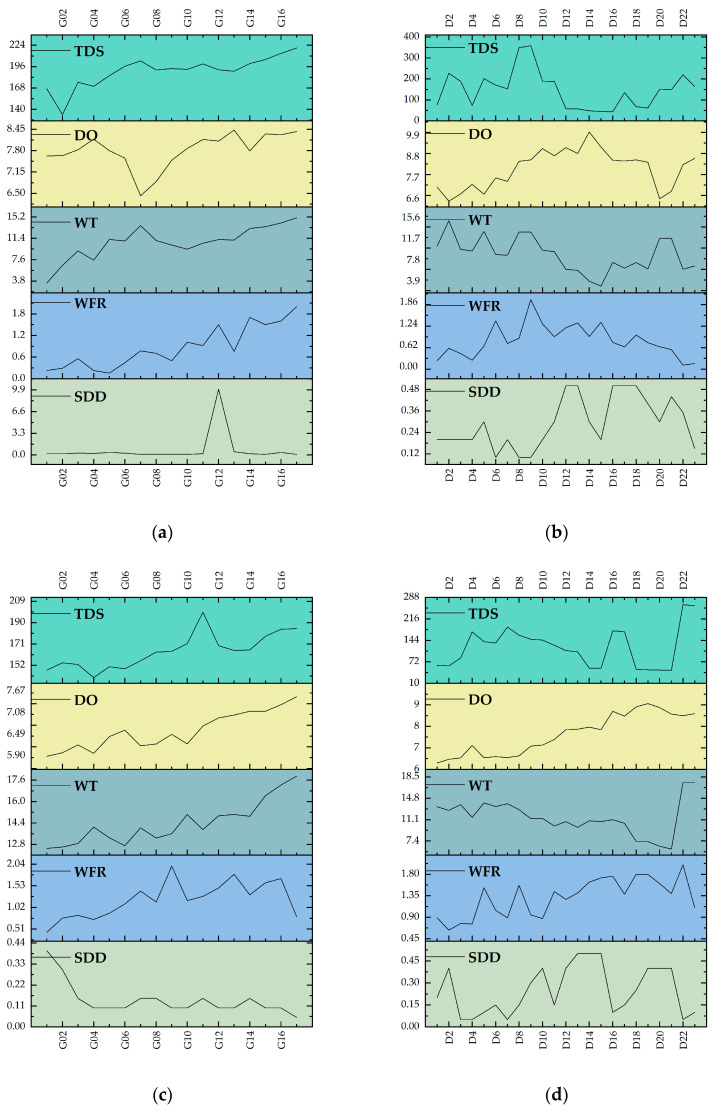
Analysis of seasonal variations in physical parameters of aquatic systems in the Yuqu River Basin: (**a**) physical parameters of the main stream in May; (**b**) physical parameters of the tributaries in May; (**c**) physical parameters of the main stream in August; (**d**) physical parameters of the tributaries in August. Abbreviations: “TDS” stands for total dissolved solids, “DO” represents dissolved oxygen, “WT” denotes water temperature, “WFR” represents water volume flow rate, and “SDD” stands for Secchi disk depth.

Physical monitoring data in the mainstem and tributaries of the Yuqu River in May and August are recorded in Figure 4. SDD showed essentially the same range of variability in the mainstem and tributaries in May, ranging from 0 to 224 in May and decreasing slightly to 209 in August. WFR showed a range of 0.0 to 1.8 in both the mainstem and the tributaries in May, while by August, the maximum rate in the mainstem had decreased slightly to 1.53. In May, the water temperature (WT) at various sites in the mainstream and tributaries exhibited fluctuations ranging from 3.8 to 15.2 °C and 3.9 to 14.7 °C, respectively. The range of WT variation between the tributaries and the mainstream was broadly similar. However, by August, the WT range in the tributaries experienced a more substantial change, whereas the WT in the mainstream remained relatively stable. DO showed some variation between May and August, with DO ranging from 0.6 to 15.2 in both dry and tributary streams in May, while it increased in the dry streams in August, reaching 17.6. TDS ranged from 140 to 224 in the mainstem and tributaries in May and decreased to 152 to 209 in the mainstem in August. Overall, the physical indicators of the tributaries and the mainstream exhibited comparable variation ranges in May, particularly for parameters such as DO, WT, and WFR. However, in August, specific parameters in the mainstream exhibited distinct seasonal variations. The inter-site differences in the physical water quality indicators of the mainstream were relatively minor, whereas those in the tributaries were more pronounced. Parameters such as TDS, WT, and DO showed considerable variation across the tributary survey sites, indicating a more marked response to seasonal environmental changes.

**Figure 5:**
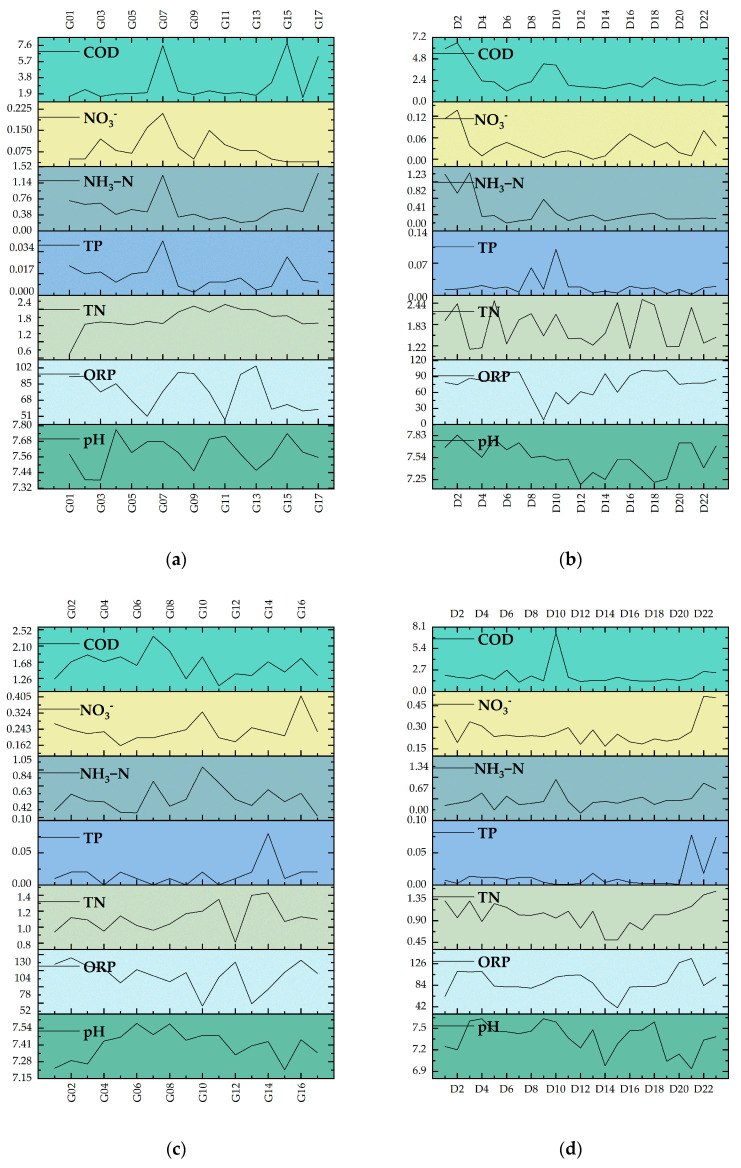
Analysis of seasonal variations in chemical parameters of aquatic systems in the Yuqu River Basin: (**a**) chemical parameters of the main stream in May; (**b**) chemical parameters of the tributaries in May; (**c**) chemical parameters of the main stream in August; (**d**) chemical parameters of the tributaries in August. Abbreviations: “COD” stands for chemical oxygen demand, “NO_3_^−^” represents nitrate ion, “NH_3_-N” denotes ammonia nitrogen, “TP” represents total phosphorus, “TN” represents total nitrogen, “ORP” stands for oxidation-reduction potential, and “pH” represents pondus hydrogen.

Figure 5 documents the chemical parameters of water quality in the mainstem and tributaries of the Yuqu River in May and August, illustrating the temporal seasonal variations in the chemical parameters across different water systems (tributaries and mainstem). The analysis reveals the similarities and differences in the trends of chemical parameter changes between the various water systems. In terms of pH, there was a decrease in both the mainstem and tributaries in May and August, with the pH range in the mainstem decreasing from 7.32 to 7.80 in May to 7.15 to 7.54 in August, while in the tributaries, it decreased from 7.25 to 7.83 to 6.9 to 7.5. The ORP increased in both the mainstem and tributaries, especially in August, when it reached 52 to 130 units, while the ORP in the tributaries increased from 42 to 126. Trends in TN and TP showed marked differences: in May, the TN values were relatively high in the mainstem and tributaries, ranging from 1.2 to 2.4 and 1.22 to 2.44, respectively, but by August, the TN values had decreased in both streams, especially in the tributaries where they fell to 0.45 to 1.35. Similarly, TP in the tributaries was lower in May at only 0 to 0.098, increasing slightly to 0 to 0.16 in August, while TP in the mainstem declined from −0.13 to 1.52 in May to 0 to 1.05 in August. Changes in NH_3_-N showed different trends for tributaries and mainstems, with tributary NH_3_-N higher in May, ranging from 0.41 to 1.23, but declining to 0.15 to 0.45 in August; in contrast, mainstem NH_3_-N values increased from 0.11 to 0.22 in May to 0.16 to 0.40 in August. NO_3_^−^ and COD also showed different patterns of change in the tributaries and mainstem in the two months, especially in the mainstem, where there was a significant increase in NO_3_^−^ to 0.162 to 0.405 in August, while in the tributaries, the change was more moderate. Overall, water quality parameters fluctuated more in the tributaries, reflecting more dramatic hydrological changes. In contrast, water quality changes in the mainstem were relatively stable in May and August, showing more consistent water quality dynamics.

**Figure 6:**
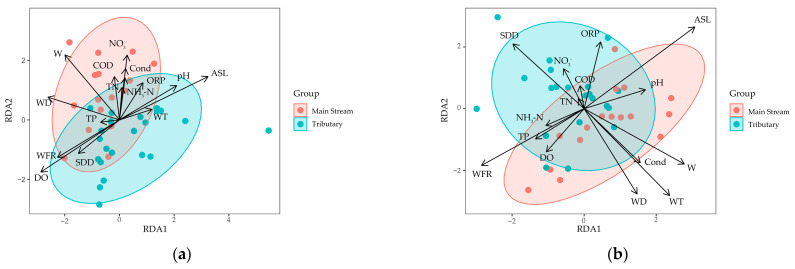
Redundancy analysis (RDA) between dominant species of macrobenthos and environmental factors in different seasons in Yuqu River Basin: (**a**) dry season; (**b**) wet season. Abbreviations: “ASL” represents altitude; “W” denotes stream width; “WD” stands for water depth; “WFR” indicates water volume flow rate; “SDD” is the abbreviation for Secchi disk depth; “WT” represents water temperature; “pH” signifies pondus hydrogenii; “DO” stands for dissolved oxygen; “Cond” denotes conductivity; “ORP” is the abbreviation for oxidation-reduction potential; “TN” represents total nitrogen; “TP” stands for total phosphorus; “NH_3_-N” indicates ammonia nitrogen; “NO_3_^−^” represents nitrate ion; and “COD” is the acronym for chemical oxygen demand.

The RDA results for the dry season (Figure 6a) indicate that the first two axes (RDA1 and RDA2) explain the majority of the variation in species–environment relationships. Key environmental factors influencing the macrobenthos community include dissolved oxygen (DO), elevation (Ele), width, and depth. Among these, DO was the most influential variable, showing a strong negative correlation in both RDA1 and RDA2 (*R*^2^ = 0.3772, *p* = 0.001), suggesting that oxygen availability plays a critical role in shaping macrobenthos community structure during the dry season.

Elevation, width, and depth also played significant roles in structuring the community. Elevation explained a significant portion of the variance along RDA1 (*R*^2^ = 0.4176, *p* = 0.001), while width and depth influenced community structure along RDA2 (width: *R*^2^ = 0.3138, *p* = 0.002; depth: *R*^2^ = 0.2527, *p* = 0.007). Additionally, nitrate (NO_3_) was a significant factor in the dry season (*R*^2^ = 0.1744, *p* = 0.041), indicating that nutrient levels, particularly nitrogen compounds, have a greater effect on macrobenthos communities during periods of lower water flow. pH was also a significant factor (*R*^2^ = 0.1895, *p* = 0.023), suggesting that changes in water acidity influenced species composition during this period. Other environmental variables, such as temperature, conductivity, and ORP, had less influence during the dry season. Notably, temperature lost its significance (*p* = 0.405), contrasting with its stronger role in the rainy season.

The RDA plot (Figure 6b) for the rainy season highlights significant relationships between macrobenthos communities and environmental factors, including elevation, width, velocity, temperature, and water clarity. Elevation exerted a strong positive influence on species distribution along RDA1 (*R*^2^ = 0.5322, *p* = 0.001), highlighting its key role in structuring macrobenthos communities during the rainy season. Width and velocity also had significant effects on community distribution (width: *R*^2^ = 0.3762, *p* = 0.001; velocity: *R*^2^ = 0.3745, *p* = 0.001), particularly along RDA2. Other important factors included temperature (*R*^2^ = 0.4609, *p* = 0.002), which strongly influenced the second axis, and depth (*R*^2^ = 0.3291, *p* = 0.002), particularly in tributaries with shallower waters. Variables such as conductivity and ORP were marginally significant, with *p*-values of 0.036 and 0.054, respectively, suggesting a smaller but still notable influence on macrobenthos communities. Dissolved oxygen (DO), total nitrogen (TN), and chemical oxygen demand (COD) were not significant predictors of species distribution during the rainy season.

### 3.3. Interactions Among Environmental Factors

**Figure 7:**
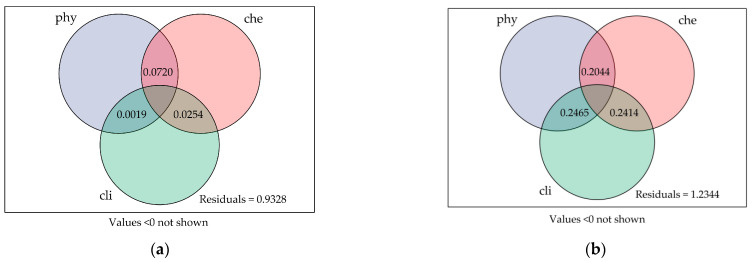
Main influencing factors and their contributions to macrobenthos community structure changes in different seasons in Yuqu River Basin: (**a**) dry season; (**b**) wet season. Abbreviations: “phy” represents the physical parameters of the water, “che” represents the chemical parameters of the water, and “cli” represents climatic factors.

In the May VPA analysis (Figure 7 and Table S3), the decomposition of variation in macrobenthos community structure in the Yuqu River Basin in terms of the dimensions of three major environmental factors, namely, hydrophysical (phy), hydrochemical (che), and climatic (CLI), was investigated. The results showed that the hydrophysical factor played a significant role in explaining the variation in community structure, with an explained variance of 0.0820. This suggests that hydrophysical factors, such as water depth and flow velocity, influence the formation and maintenance of macrobenthos community structures during the month. In addition, water chemistry factors had an explanatory power of 0.0720 for community variation, suggesting that chemical characteristics, such as dissolved oxygen, pH, and nutrient concentrations in the water, also influenced the composition and distribution of macrobenthos communities, albeit slightly less than hydrophysical factors. The explanatory power of climatic factors on community structure was 0.0254, which means that climatic conditions, such as temperature and precipitation, had a relatively small effect on the macrobenthos community in May, which is a relatively dry time in the Yuqu River Basin. Climatic factors had limited influence on the water environment at this time of the year and thus made a smaller contribution to the variability in the macrobenthos community.

The value of the residuals in the decomposition of variation for that month was 0.9328, indicating a significant amount of community structure variation that was not explained by factors in the hydrophysical, hydrochemical, and climatic dimensions. The presence of residuals may suggest that other factors not yet included play an essential role in influencing macrobenthos community structure, such as substrate type, biological interactions, and human activities. In addition, the graph indicates that values below zero are not shown, suggesting that there is no negative amount of explanation in the variance interpretation, which helps to ensure the credibility and scientific validity of the results of the analyses. Overall, the May VPA analyses showed that hydrophysical and hydrochemical factors were the main drivers in explaining variation in macrobenthos community structure, while climatic factors had a weaker effect.

In the August VPA analysis, the variance in the macrobenthos community structure was also decomposed for the three dimensions of hydrophysical, hydrochemical, and climatic factors. Still, the results showed significant differences from those of May, the rainy season in the Yuqu River Basin, with increased precipitation, which had far-reaching impacts on river hydrological characteristics and ecosystems. This month, the amount of variance explained by hydrophysical factors on community structure increased to 0.2044, indicating that the importance of hydrophysical factors increased significantly during the rainy season. The variations in water depth and flow velocity have significantly impacted the habitat of macrobenthic organisms, leading to corresponding alterations in the community structure in response to changes in the ecological environment.

The amount of variance explained by the water chemistry factor was 0.2414 in August, which was significantly higher than in May, showing that changing water chemistry conditions on macrobenthos fauna were more pronounced during the rainy season. Precipitation leads to an increase in nutrient and organic matter inputs into the watershed, thus altering the nutrient composition and concentration of dissolved substances in the water, and these changes directly affect the survival and distribution of macrobenthos fauna. Therefore, the influence of water chemistry factors on macrobenthos community structure becomes more important in August. Most notably, the climate factor explained 0.2465 of the macrobenthos community structure in August, which was the most significant contribution of the three factors. This suggests that during the rainy season, climatic factors, such as fluctuations in temperature and precipitation, have a significantly more substantial effect on macrobenthos fauna. Climatic changes during the rainy season significantly impacted water temperature, flow velocity, and water column nutrients, all of which further influenced the composition and distribution of the macrobenthos community.

The residual value of 1.2344 for August, although it still exhibited a large amount of unexplained variation, was an increase over the residual for May, possibly due to the higher complexity of environmental factors during the rainy season, which resulted in a more difficult amount of variation to explain. In this case, unincorporated factors, such as anthropogenic disturbances, biotic interactions, and macrobenthos lifecycles, may have influenced changes in community structure to a greater extent. Overall, the August VPA analyses showed that environmental factors had significantly more power to explain variation in community structure, with climate, water chemistry, and water physics factors having relatively balanced effects on macrobenthos communities.

## 4. Discussion

### 4.1. Spatiotemporal Distribution Characteristics of Macrobenthos Animal Communities

The natural flow of tributaries is vital for preserving the biodiversity of riverine aquatic life. As a key first-order tributary to the middle reaches of the Nujiang River, the Yuqu River’s maintenance of its natural flow state supports a diverse aquatic habitat in these riverine areas [40,41]. This study documented 102 taxonomic units of macrobenthic organisms from the Yuqu River’s mainstream and tributaries, indicating a high level of biodiversity. Species composition shows that aquatic insects, which thrive in running waters and signify clean conditions, such as Ephemeroptera, Plecoptera, Trichoptera, and Diptera, are dominant across the Yuqu River Basin. This dominance suggests that the river offers an exceptional habitat for sensitive aquatic insects in the Nujiang River’s middle reaches. Given the extensive dam construction and hydropower development in China’s southwestern region, which can influence macrobenthic community composition and distribution [42], investigating species diversity in the natural state of the Yuqu River Basin is crucial for conserving aquatic biodiversity in the Nujiang River’s middle reaches and for safeguarding aquatic life in southwestern China. This research also serves as a scientific foundation for future studies on the biodiversity composition of high-altitude river systems.

Temporal distribution patterns in the Yuqu River Basin show that aquatic insects, especially those in the orders Ephemeroptera, Plecoptera, Trichoptera, and Diptera, dominate both dry and rainy seasons. However, macrobenthic diversity varies seasonally, with higher species richness but lower abundance in the dry season and vice versa during the rainy season. This aligns with numerous studies on seasonal effects on macrobenthos. For instance, dos Santos and colleagues’ research on the Amazon’s Argochoa Island revealed that seasonal hydrological changes impact macrobenthic diversity and abundance, with flooding during the rainy season often suppressing species diversity [43]. In their investigation of the Wei River in China, Zhang et al. posited that the abundance and biomass of macrobenthic communities in the tributaries consistently remained lower than those observed in the mainstem of the river [44]. In this study, the number of species of macrobenthos in the tributary sections exhibited only minor differences compared to the mainstem. However, individual counts of macrobenthos were significantly higher in tributaries than in the mainstem. This contrast may be due to the unique hydrological and topographical conditions of the Yuqu River Basin. The basin’s varied altitudes create distinct habitats in the tributaries. These areas, with stable flow velocities, narrower river widths, and abundant substrates like roots and stones, are more favorable for macrobenthic growth and reproduction, explaining the observed distribution differences. This is particularly evident in the species distribution of arthropods, annelids, and platyhelminths. These findings are consistent with the conclusions of Nautiyal et al. [45]. The interaction of factors like flow velocity, water depth, and habitat conditions, especially the complex topography and varied hydrodynamics in tributary reaches, is a key factor in the distinct ecological environments of the Yuqu River Basin. This interaction significantly shapes the ecological niches of macrobenthic communities. Additionally, when examining the composition and abundance of macrobenthos, it is crucial to consider the interaction between abiotic factors and biological interactions. While this study has discussed the response of macrobenthic community structure to environmental changes by analyzing the seasonal fluctuations in species richness and individual numbers of macrobenthic organisms, the impact of multivariate factors, such as abiotic and biological interactions, on the research outcomes necessitates further in-depth investigation in the future.

### 4.2. Analysis of Environmental Factors Affecting the Structure of Macrobenthos Animal Communities

Dissolved oxygen (DO), altitude, river width, and river depth are pivotal factors affecting community structure, which is a finding that aligns with numerous studies conducted in low-altitude rivers. For instance, Rigaud et al. discovered in their research on the Berre Lagoon that DO is a crucial factor influencing the composition of macrobenthic communities, particularly for those species with higher oxygen demands. A reduction in the dissolved oxygen content in the water body may lead to significant alterations in the structure of these communities [46]. However, the present study revealed an inverse correlation between the number of species of macrobenthic organisms and water flow velocity, as well as DO, which contrasts with the findings of the aforementioned studies. This discrepancy may be associated with the specific composition of the macrobenthic community in the Yuqu River Basin. The survey identified that the macrobenthic community in the Yuqu River Basin is predominantly composed of Diptera and chironomid larvae, which primarily depend on cutaneous respiration and are largely burrowing organisms with lower oxygen demands. The high-velocity water flow not only increases their energy expenditure but also raises the dissolved oxygen content in the water, thereby imposing stress on their survival [47]. The effects of changes in dissolved oxygen on macrobenthos invertebrates have been a topic of interest in various studies [48,49]. For example, a study by Gómez et al. found that low dissolved oxygen levels had significant impacts on aquatic invertebrates and fish [50]. Additionally, Rakocinski et al. observed that despite the remarkable ecological resilience exhibited by macrobenthos in conjunction with the decline in dissolved oxygen concentrations during the summer, there was a significant reduction in the total density and productivity of the macrobenthos community [51]. Furthermore, the study by Chapman, D.V. et al. highlighted the importance of monitoring water quality conditions, such as low dissolved oxygen, to identify pollutants responsible for impacts on macrobenthos [52]. In contrast, a study by Kim et al. did not find consistent effects of dissolved oxygen concentration on macroinvertebrate diversity. However, they noted that other factors, such as temperature, may also play a role in influencing macroinvertebrate richness [53]. Additionally, the study by Koperski found that nutrient enrichments had both linear and nonlinear effects on the diversity of macrobenthos invertebrates, suggesting that multiple factors need to be considered when studying the effects of changes in dissolved oxygen on macrobenthos invertebrates [54]. Overall, dissolved oxygen levels can have significant impacts on macrobenthos invertebrates, with implications for their diversity and community structure [55,56].

This study found that the concentration of nitrate (NO_3_^−^) in the water body during the dry season was significantly lower than that in the rainy season, while the number of species of macrobenthic organisms was significantly higher in the dry season. This suggests that the growth and reproduction of macrobenthic organisms may be influenced by the concentration of NO_3_^−^ in the water, which is consistent with the findings of Yeanny et al. [57]. The pronounced disparity in NO_3_^−^ concentrations within the Yuqu River watershed between the dry and rainy seasons can be ascribed to the distinctive agricultural and livestock practices prevalent in the area. The extensive application of organic fertilizers, in conjunction with the augmented precipitation during the rainy season, results in the buildup of NO_3_^−^ within the water bodies. This buildup has the potential to disrupt the equilibrium of the aquatic ecosystem. Despite high nitrate concentrations in lowland, intensively farmed streams, these levels did not significantly impact macrobenthic invertebrate communities, likely due to the depletion of sensitive species. Nevertheless, macrobenthic macrofauna, such as *Limecola balthica*, *Marenzelleria* spp., and *Monoporeia affinis*, are pivotal in nitrogen cycling and the functioning of the ecosystem. These macrofauna holobionts contribute to macrobenthos metabolism, nutrient recycling, and dissimilative nitrate reduction [58,59]. In marine mangroves, nitrate reduction rates increased with nitrate concentration and organic carbon load, highlighting the potential of mangrove sediments to mitigate nitrogen pollution [60]. In coastal mariculture zones, seasonal deoxygenation enhanced denitrification and dissimilatory nitrate reduction to ammonium, impacting macrobenthos nitrate reduction pathways and microbial communities [61]. Dead zones caused by eutrophication lead to hypoxic conditions that disrupt macrobenthos communities, decrease species diversity, and result in mass mortality events [62]. Overall, macrobenthos communities are sensitive to changes in nitrate concentrations, organic carbon load, and oxygen levels, highlighting the importance of understanding these factors for ecosystem health and management.

Hydrological changes during the wet season significantly altered the drivers of community structure compared to the dry season. Elevation, river width, flow velocity, and water temperature were key influencers, with flow velocity particularly impacting the spatial distribution of macrobenthos fauna during the rainy season [9,45,63]. This aligns with the findings of Beermann et al. in stream mesocosm studies, in which they observed a trend of decreased habitat quality and species density and richness in macrobenthos communities with increased variability in flow velocity. Specifically, there was a negative synergistic effect on the abundance of species such as mayflies Ephemeroptera and chironomids, which are particularly sensitive to flow alterations [64]. This study reveals a significant altitude gradient in the Yuqu River Basin, resulting in distinct hydrological conditions like water temperature and flow velocity between upstream and downstream areas. The rainy season’s increased precipitation accelerates water flow, profoundly affecting macrobenthic growth and reproduction, especially for Diptera burrow-dwellers. Conversely, species adapted to water flow have maintained stability, a finding that is consistent with previous research.

### 4.3. Impact of Seasonal Hydrological Changes on the Adaptive Strategies of Macrobenthos Animal Communities in the Yuqu River Basin

The structure of macrobenthic communities is tightly linked to the water conditions in their homes. These conditions, including water’s physical and chemical traits, food sources, and other environmental aspects, can vary across different water systems and seasons. These variations can directly or indirectly influence the growth, breeding, and community changes in macrobenthic organisms. Over time, these shifts result in changes to the species makeup and community structure of macrobenthic organisms in the area [65,66]. The RDA analyses showed that different environmental factors significantly drove the macrobenthos community structure in the Yuqu River Basin during the dry (May) and rainy (August) seasons. This seasonal difference reflects the sensitivity of macrobenthos fauna to dynamic changes in environmental factors and their survival adaptation strategies under different ecological pressures. Specifically, changes in the macrobenthos community during May were mainly associated with changes in the depth and width of the water column and nutrients (e.g., total nitrogen and total phosphorus), and these factors had a particularly significant effect on the community during the dry season when the environment was relatively stable. This finding is essentially in concordance with the research outcomes presented by Yan and Zhang et al. [67,68]. In the dry season, when the river volume is relatively small and the water body environment is more stable, the habitat of macrobenthos fauna mainly depends on the physical characteristics of the water body. These conditions collectively determine the efficiency of macrobenthic communities in acquiring nutritional resources within the aquatic environment and their adaptive mechanisms to the physical surroundings. During May, which falls within the dry season, the high-altitude regions experience lower temperatures and precipitation, resulting in reduced meltwater from snow-capped mountains, slower water flow rates, and a relatively lower input of terrestrial organic matter due to the scarcity of precipitation. In response to this environmental stress, the macrobenthic community exhibits specific adaptive strategies to maintain ecological balance in the water body [69]. Specifically, the structural composition of the macrobenthic animal community may shift toward species that are adapted to low water flow velocities and low temperatures and capable of tolerating low nutrient levels. During the dry season, the species diversity of freshwater insects belonging to the order Trichoptera is relatively high, whereas it significantly decreases during the rainy season. Notably, during the rainy season’s surveys, some species from families such as Leptoceridae, Lepidostomatidae, and Hydropsychidae were not observed. On one hand, the larvae of these large benthic insects are nest-building and primarily feed on organic detritus, bacteria, and algae. In the rainy season, with faster water flow, the larvae face increased difficulty in obtaining food, and their nests are more challenging to secure, which may affect their survival and reproduction. On the other hand, the large benthic insects of the families Leptoceridae, Lepidostomatidae, and Hydropsychidae typically enter the pupal stage and emerge during the summer months [70,71,72], with August being the peak of this season. Many of these insects have already emerged onto land by August, resulting in a substantial reduction in their numbers in the water bodies. However, in this survey, it was found that families such as Brachycentridae, Glossosomatidae, and protochironomidae were still widely distributed in August. This may be related to their unique adaptive strategies, such as extended or shortened emergence cycles, enhanced feeding abilities, and adaptations to high water velocities. However, the specific mechanisms require further in-depth research in the future.

Recent scholarly investigations have demonstrated that physical attributes, such as the depth and breadth of water bodies, along with the concentration of nutrients, have become pivotal determinants in shaping the structure of aquatic communities [63,67]. This means the macrobenthos community structure depends more on the physical environment, especially under stable water temperatures and flow rates. Such stable physical conditions favor the survival and reproduction of species that can adapt to low flow velocities and efficiently use limited nutrients. In addition, the concentration of nutrients (e.g., total nitrogen, total phosphorus) also impacts community structure, especially when nutrient inputs are low, and macrobenthos fauna must maintain their survival by increasing resource use efficiency [67,68]. Unlike the dry season, the August RDA results showed a significant increase in the effects of climatic factors (e.g., temperature) and factors related to hydrodynamics (e.g., current velocity and water turbidity) on community structure. This change was mainly attributed to the drastic changes in the water body environment due to heavy precipitation during the rainy season [73]. During the rainy season, as precipitation increases, river flow velocity increases significantly, water levels rise, and water bodies become more turbid. These changes profoundly affect the survival and distribution of macrobenthos fauna. In particular, the changes in hydrodynamics directly affected the habitat stability of macrobenthos and increased habitat heterogeneity, making it necessary for macrobenthos to have a strong adaptive capacity to cope with these changes [74]. Moreover, heavy rainfall during the rainy season alters the water body’s physical traits. It introduces substantial nutrients, such as nitrogen and phosphorus, through runoff. This influx of nutrients has significantly altered water chemistry, enabling species capable of thriving in high-nutrient conditions to rapidly dominate the community. Consequently, the macrobenthic community composition has changed dramatically, with certain species quickly adapting to these enriched environments and proliferating during the rainy season. Simultaneously, changes in water temperature have also directly affected the macrobenthos, potentially enhancing the metabolic activity and growth rates of some species, thereby altering the community’s overall structure [75]. In this study, the diversity and abundance of arthropods and platyhelminths were significantly higher during the rainy season compared to the dry season, indicating a notable ecological adaptability of these species to high-flow water environments. Nevertheless, this finding does not rule out the potential influences of anthropogenic factors and interspecies interactions, which may also have objective impacts on their survival and reproduction.

Comparing RDA results between May and August illustrates how seasonal climate variations dynamically alter macrobenthos community structure via physical and chemical processes. In the dry season, the stable water column makes macrobenthos more reliant on physical habitat features. Conversely, the rainy season’s climatic changes induce more dynamic water conditions, with increased current velocities and nutrient inputs collectively influencing species competition and resource distribution. The variable environment of the rainy season necessitates a rapid response from macrobenthos fauna to adapt to shifting hydrological and chemical conditions. This swift adaptability is a crucial mechanism enabling the macrobenthos to thrive in extreme conditions [76]. Seasonal fluctuations expose the mechanisms by which macrobenthos communities cope with environmental variability. In the stable dry season, populations are sustained by adapting to habitat features and enhancing nutrient efficiency. This seasonality also highlights the adaptation strategies of macrobenthic species. The dry season’s stable conditions, which are evidenced by the prevalence of annelids and the higher species richness relative to the rainy season, suggest niche differentiation. Annelids’ competitive edge could stem from their efficient use of substratum resources. However, during the rainy season’s rapid environmental shifts, macrobenthos must exhibit greater adaptability to cope with changing habitats and competitive challenges [42].

### 4.4. Driving Mechanisms of Seasonal Environmental Changes on the Structural Dynamics of Macrobenthos Animal Communities in the Yuqu River Basin

Variations in the role of environmental variables across seasons are a well-established phenomenon in freshwater ecology. Seasonal changes often result in significant shifts in water’s physical and chemical properties, making it essential to analyze these trends within the framework of seasonal dynamics. In such regions, temperature may not be the sole determining factor; changes in precipitation often have a more pronounced effect on the chemical properties of water and macrobenthic communities [77]. For instance, the temperature may lose significance during the dry season due to minimal variation. Still, it becomes more critical during the rainy season, possibly because of increased water turbidity or shifts in thermal stratification that influence species distribution [77,78]. The effects of variations in water flow and temperature on macrobenthic organisms are often interactive. As temperature increases, dissolved oxygen (DO) in the water decreases, thereby worsening the habitat conditions for macrobenthic microorganisms [79]. The effect is most pronounced during the dry season, when reduced water flow intensifies its impact. Dissolved oxygen (DO) is a significant predictor in the dry season but loses significance in the rainy season, possibly due to increased flow and oxygenation. A VPA analysis reveals a significant difference in the macrobenthic community structure in the Yuqu River Basin between the dry season (May) and the rainy season (August). In May, physical and chemical water parameters are key drivers of macrobenthic growth and reproduction, with climatic factors playing a lesser role due to the river’s stable environment. In the rainy season, factors like water depth, flow velocity, and nutrient availability may limit macrobenthic habitats, particularly for nesting species like Trichoptera and surface-dwelling Diptera larvae. The distribution of macrobenthos is primarily determined by their ability to adapt to habitat characteristics, such as nutrient acquisition and tolerance to flow, which dictates their survival strategies in low-nutrient, stable-flow conditions [80,81]. This also explains the low influence of climatic factors during the dry season; stable climatic conditions have a relatively weak regulatory effect on the physical and chemical properties of the water column. In addition, the research process may be beset by certain variables that need to be adequately accounted for. For example, the impact of human activities on aquatic ecological environments can subsequently affect the structure and composition of macrobenthic communities. During the dry season, activities such as grazing, riverbank construction, and industrial development may accumulate a significant amount of pollutants. When the rainy season arrives, substantial rainfall can carry these pollutants from the land into the aquatic ecosystem, leading to alterations in the physicochemical parameters of the water body; this could be a possible reason for the higher variance in VPA analysis during May and August. These changes may indirectly influence the structure and composition of macrobenthic animal communities, and these factors may contribute to the incomplete explanation of certain discrepancies in community structure dynamics. Furthermore, within aquatic ecosystems, the interactions among various populations form a complex network, encompassing competitive relationships, predatory interactions, symbiotic associations, and disruptive relations among macrobenthic organisms, among others. This network includes numerous uncontrollable factors that collectively influence the structure and function of the community. Hence, although variance partitioning analysis (VPA) is an effective tool for quantifying the impact of different factors on community dynamics, its explanatory power may be limited in practical applications. This is because the dynamic changes in aquatic ecosystems are often subject to the combined effects of multiple factors, including but not limited to seasonal climatic variations, hydrological cycles, nutrient inputs, and human activities. The interplay among these factors can lead to increased uncertainty in predictive models, thereby constraining the accuracy and reliability of VPA analysis. Future studies should consider these omitted variables more profoundly and employ more sophisticated methodologies to investigate the response mechanisms of macrobenthic communities to environmental changes.

By comparing the results of May and August, it can be seen that seasonal variations significantly affect the structure of the macrobenthos community by altering the water body’s physical, chemical, and climatic conditions. In May, the environment was relatively stable, and the macrobenthos relied on the ability to adapt to the physical and chemical conditions for survival. However, influenced by climatic changes, the marked increase in temperature and the alterations in precipitation frequency and intensity from May to August have prompted a significant shift in the structure of the macrobenthic community. This shift underscores the sensitivity of macrobenthic organisms to seasonal climatic fluctuations and highlights their dynamic adaptive strategies in response to such environmental heterogeneity. For instance, during August (the rainy season), there was a significant decrease in the number of species of macrobenthic Diptera, which may be attributed to the impact of precipitation. The water flow velocity increased dramatically in August, and Diptera was primarily represented by burrowing macrobenthic organisms that are highly sensitive to changes in water flow. Nevertheless, despite the reduction in number of species, the individual count still exceeded that of May. This suggests that certain arthropod species, such as Ephemeroptera and Trichoptera, adopt a strategy of increasing individual abundance to cope with the ecological conditions of rapid water flow. This could be a survival mechanism of macrobenthic organisms to maintain the stability of the ecosystem. Especially in extreme highland environments where macrobenthos have to cope with drastically changing hydrological and chemical conditions, their community structure is seasonal dynamics that directly reflect the organisms’ adaptation to environmental stresses and resource utilization [82]. In the present study, during the rainy season, there was an observed increase in nutrient concentrations within the aquatic environment, which was concurrent with a marked decrease in the diversity of arthropods. Nevertheless, the individual count of these organisms was maintained at a relatively high level. This suggests that specific macrobenthic populations rapidly increase and achieve a dominant status during the rainy season. By enhancing the efficiency of nutrient utilization within the community, these populations contribute to maintaining ecosystem stability. This phenomenon may be indicative of a self-restorative mechanism within the ecosystem.

Meanwhile, the significant effects of climatic factors also implied the critical regulatory role of factors, such as water temperature and precipitation, on the distribution and population dynamics of macrobenthos fauna in extreme environments [83]. These results suggest that macrobenthos communities in the Yuqu River Basin exhibit different survival and adaptive strategies between the dry and wet seasons. The mechanisms behind them include responsiveness to environmental changes, resource competition, and intra-community interactions. This study has provided a scientific basis for subsequent research on the biodiversity of high-altitude river ecosystems, as well as for the management of these ecosystems and the conservation of their biodiversity.

## 5. Conclusions

The findings based on the macrobenthic community structure in the Yuqu River Basin reveal distinct spatiotemporal variations between the dry and wet seasons. During the dry season, with relatively stable water temperatures and the river in a circulation state, the impact of physicochemical indices of the water body (such as dissolved oxygen, ammonia nitrogen, nitrate, total nitrogen, and total phosphorus) on the macrobenthic community structure is more pronounced. This suggests that during this period, the growth, reproduction, and distribution of macrobenthic organisms are less influenced by physical habitat conditions and are primarily constrained by changes in the physicochemical indices of the water body and the stability of nutrient sources.

In contrast, during the wet season, climatic factors (such as temperature) and hydrodynamic-related factors (such as flow velocity and water turbidity) exert a more significant influence on the community structure. The increased water flow velocity and water level resulting from heavy precipitation lead to drastic changes in the habitat environment, enhancing habitat heterogeneity. These hydrodynamic changes provide an advantage to species that can rapidly adapt to high nutrient loads and fluctuations in water flow, resulting in a reconfiguration of the community structure. This phenomenon indicates that the macrobenthic organisms in the Yuqu River Basin demonstrate a strong capacity for environmental adaptation in the face of seasonal environmental changes, particularly in response to variations in hydrology and nutrient input.

Overall, this study revealed that the spatial and temporal dynamics of macrobenthos communities in the Yuqu River Basin are significantly driven by environmental factors, especially in the highly high-altitude environment, where changes in hydrological and climatic factors play a decisive role in community structure. However, it is imperative to account for the integrated influence of multifaceted factors, such as anthropogenic factors, interspecific interactions, and the unique adaptive strategies of organisms to their environment on the structure of biological communities.

## Figures and Tables

**Table 1:** Species number and individual number composition of macrobenthos in Yuqu River Basin. Abbreviations: “SP” represents the number of species of macrobenthic animals; “N” represents the number of individuals of macrobenthic animals.

Item	Arthropoda		Annelida		Mollusca		Platyhelminthes	
	SP	N	SP	N	SP	N	SP	N
**dry season**	90	1424	5	60	3	3	1	3
**rainy season**	51	1632	2	160	2	3	1	34
**main river**	92	1100	6	45	3	6	1	2
**tributary**	84	1956	6	175	0	0	1	35

## Data Availability

The data supporting this study’s findings are available from the corresponding authors upon reasonable request.

## Supplementary Materials

The following supporting information can be downloaded at https://www.mdpi.com/article/10.3390/biology14020120/s1: Table S1: Graphical representation of altitudinal gradient and seasonal variations in width and depth at sampling sites within the Yuqu River watershed, Table S2: Macrobenthic faunal inventory of the Yuqu River Basin, Table S3: Redundancy analysis (RDA) results predicting macroinvertebrate species composition using selected environmental variables.
