# Supplementary material for: Seasonal Variations in Macrobenthos Communities and Their Relationship with Environmental Factors in the Alpine Yuqu River
Source: Biology (Basel). 2025 Jan 24;14(2):120. doi: 10.3390/biology14020120 (PMC11852101; doi:10.3390/biology14020120)
Supplement: Supplementary file 1 [file biology-14-00120-s001.zip › biology-3402076-supplementary.pdf]

Table S1. Graphical Representation of Altitudinal Gradient and Seasonal Variations in Width and Depth at Sampling Sites within the Yuqu River Watershed. Abbreviations: "ASL" represents altitude; "W" denotes stream width; "WD" stands for water depth.

stemflow	ASL (m)	season	WD (m)	W (m)	tributary	ASL (m)	season	WD (m)	W (m)
G01	4595	dry season	0.2	8	D1	4351	dry season	0.2	6.5
		rainy season	0.4	14.2			rainy season	0.2	20.8
G02	4419	dry season	0.3	19.6	D2	4424	dry season	0.4	4.5
		rainy season	0.3	16.8			rainy season	0.4	15.5
G03	4312	dry season	0.5	51	D3	4150	dry season	0.4	6
		rainy season	1.2	38.5			rainy season	0.3	6
G04	4130	dry season	0.25	30	D4	4092	dry season	0.2	10
		rainy season	2	54.2			rainy season	0.7	5.8
G05	4123	dry season	0.4	36.8	D5	3814	dry season	0.3	5
		rainy season	1.5	47.9			rainy season	0.3	8.4
G06	4089	dry season	0.5	70	D6	3983	dry season	0.3	4
		rainy season	1.5	63.6			rainy season	0.4	8.5
G07	3959	dry season	0.7	30.4	D7	4239	dry season	0.3	4
		rainy season	1.5	64.3			rainy season	0.6	21.4
G08	3794	dry season	2	30	D8	4216	dry season	0.5	80
		rainy season	2	32.9			rainy season	0.8	21.6
G09	3805	dry season	1.2	37.5	D9	4020	dry season	0.4	60
		rainy season	3	78.6			rainy season	0.3	17.8
G10	3499	dry season	0.7	43	D10	4013	dry season	0.6	6
		rainy season	2	61.6			rainy season	0.5	9.1
G11	3410	dry season	2	31	D11	3799	dry season	0.6	4
		rainy season	1.7	46.5			rainy season	0.5	6.3
G12	3061	dry season	3	35	D12	3405	dry season	0.5	2
		rainy season	2	36.1			rainy season	0.6	12.1
G13	2833	dry season	3	28	D13	3457	dry season	0.6	2
		rainy season	2	35.8			rainy season	0.6	18.9
G14	2736	dry season	4	30	D14	3126	dry season	0.3	3
		rainy season	1.6	33.5			rainy season	0.4	10.6
G15	2277	dry season	4	40	D15	3310	dry season	0.2	3
		rainy season	1.5	34.3			rainy season	0.5	10.4

G16	2181	dry season	5	32	D16	2434	dry season	0.5	7
		rainy season	1.3	41.4			rainy season	0.7	11.6
G17	1894	dry season	2.3	24	D17	2507	dry season	0.6	8
		rainy season	2	66.8			rainy season	0.6	8.9
					D18	2907	dry season	0.5	3
							rainy season	0.251	11.9
					D19	2912	dry season	0.4	8
							rainy season	0.4	2.4
					D20	3128	dry season	0.2	17
							rainy season	0.4	3
					D21	3472	dry season	0.6	6
							rainy season	0.4	3
					D22	2150	dry season	0.35	9.8

Table S2. Macrobenthic Faunal Inventory of the Yuqu River Basin.

Phylum	Class	Order	Family	Genus	Species	dry season	rainy season
**Platyhelminthes**	**Turbellaria**	**Tricladida**	**Dugesiidae**	*Planaria*	*Dugesia japonica*	+	+
**Annelida**	**Oligochaeta**	**Tubificida**	**Tubificidae**	*Limnodrilus*	*Limnodrilus hoffmeisteri*	+	+
				Tubifex	*Tubifex tubifex*	+	
	Hirudinida	***Arhynchobdellida***	***Erpobdellidae***	*Erpobdella*	*Erpobdella* sp.	+	+
		**Gnathobdellida**	**Salifidae**	*Barbronia*	*Barbronia weberi*	+	
		**Rhynchobdellida**	**Piscicolidae**	*Piscicola*	*Piscicola geometra*	+	
**Arthropoda**	***Insecta***	***Odonata***	***Gomphidae***		*Gomphidae* spp.	+	
		Ephemeroptera	***Heptageniidae***	*Heptagenia*	*Heptagenia* sp.	+	+
				Caucasiron	*Caucasiron sp.*	+	
				Epeorus	*Epeorus* sp.	+	+
				Rhlthrogena	*Rhlthrogena* sp.	+	+
				Cinygmula	*Cinygmula sp.*	+	
			Siphlonuridae	*Siphluriscus*	*Siphluriscus* sp.	+	+
			**Ephemeridae**	*Ephemera*	*Ephemera orientalis*	+	+
					Ephemera sp.	+	
			Baetidae	*Baetis*	*Baetis* sp.	+	+
				Baetiella	*Baetiella* sp.	+	
			**Siphlonuridae**	*Siphlonurus*	*Siphlonurus sp.*	+	
			**Ephemerellidae**	*Drunella*	*Drunella bella*	+	+
				Ephemerella	*Ephemerella* sp.	+	+
				Cincticostella	*Cincticostella sp.*	+	
				Serratella	*Serratella sp.*	+	
			**Neoephemeridae**	*Neoephemera*	*Neoephemera projecta*	+	+
		Plecoptera	***Peltoperlidae***	*Peltoperlopsis*	*Peltoperlopsis* sp.	+	+

		Nemouridae	*Amphinemura*	*Amphinemura* sp.	+	+
			Nemoura	*Nemoura* sp.	+	
		Perlidae	*Togoperla*	*Togoperla* sp.	+	+
			Tetropina	*Tetropina* sp.	+	
			Paragnetina	*Paragnetina* sp.	+	
		Choloroperlidae	*Suwallia*	*Suwallia* sp.	+	+
		Perlodidae	*Isoperla*	*Isoperla* sp.	+	+
			Stavsolus	*Stavsolus* sp.	+	
		Taeniopterygidae	*Taenionema*	*Taenionema* sp.	+	
	Trichoptera	***Brachycentridae***	*Brachycentrus*	*Brachycentrus* sp.	+	+
		Glossosomatidae	*Glossosoma*	*Glossosoma* sp.	+	+
		Hydropsychidae	*Aethalopsyche*	*Aethalopsyche* sp.	+	+
			Macrostemum	*Macrostemum* sp.	+	
			Parapsyche	*Parapsyche* sp.	+	
			Hydropsyche	*Hydropsyche* sp.	+	+
			Cheumatopsyche	*Cheumatopsyche* sp.	+	
			Ceratopsyche	*Ceratopsyche* sp.	+	
		Stenopsychidae	*Stenopsyche*	*Stenopsyche* sp.	+	
		**Rhyacophilidae**	*Rhyacophila*	*Rhyacophila nigrocephala*	+	+
			Himalopsyche	*Himalopsyche* sp.	+	
		Lepidostomatidae	*Georodes*	*Georodes* sp.	+	
		Leptoceridae	*Setodes*	*Setodes* sp.	+	
		Limnephilidae	*Limnephilus*	*Limnephilus* sp.	+	
			Pseudostenophylax	*Pseudostenophylax* sp.	+	
	Coleoptera	***Dytiscidae***	*Coptotomus*	*Coptotomus* sp.	+	+
		Elmidae	*Stenelmis*	*Stenelmis* sp.	+	+
	**Diptera**	**Tipulidae**	*Hexatoma*	*Hexatoma Eriocera*	+	+
			Antochea	*Antochea bifida.*	+	+
			Dicranota	*Dicranota* sp.	+	+
			Helius	*Helius* sp.	+	+
			Tipula	*Tipula* sp.	+	
		**Diamesinae**	*Potthastia*	*Potthastia longimana*	+	+
			Pagastia	*Pagastia lanceolata*	+	+
				Pagastia orientalis	+	
			Deamisa	*Deamisa* sp.	+	
		**Calliphoridae**	*Hemipyrellia*	*Hemipyrellia ligurriens*	+	+
		**Chironomidae**	*Monodiamesa*	*Monodiamesa nitida*	+	+
		**Dolichopodidae**		Dolichopodidae spp.	+	
		**Empididae**		Empididae spp.	+	
		**Ephydridae**		Ephydridae spp.	+	
		**Simuliidae**	*Simulium*	*Simulium xinbinense.*	+	
		Dixidae	*Meringodixa*	*Meringodixa* sp.	+	
		**Chironominae**	*Polypedilum*	*Polypedilum laetum*	+	

					Polypedilum pedestre	+	
					Polypedilum paraviceps	+	
				Paratendipes	*Paratendipes albimanus*	+	+
				Rheotanytarsus	*Rheotanytarsus* sp.	+	+
				Tanytarsus	*Tanytarsus* sp.	+	+
				Microtendipes	*Microtendipes* sp.	+	
				Cyphomella	*Cyphomella* sp.	+	
				Cladotanytarsus	*Cladotanytarsus vanderwupip*	+	+
				Stictochironomus	*Stictochironomus* sp.		+
				Cryptochironomus	*Cryptochironomus rostratus*		+
				Micropsectra	*Micropsectra* sp.	+	
			**Tanypodinae**	*Thienemannimyia*	*Thienemannimyia geijskesi*	+	+
				Zavrelimyia	*Zavrelimyia* sp.	+	+
				Procladius	*Procladius* sp.	+	
				Rheopelopia	*Rheopelopia* sp.	+	
			**Orthocladiinae**	*Cricotopus*	*Cricotopus* sp.	+	+
					Cricotopus trifascia	+	+
				Chaetocladius	*Chaetocladius piger*	+	+
				Heleniella	*Heleniella omaticollis*	+	+
				Paratrichocladius	*Paratrichocladius* sp.	+	+
				Cardiocladoius	*Cardiocladoius capucinus*	+	+
				Psectrocladius	*Psectrocladius* sp.	+	
				Eukiefferiella	*Eukiefferiella elkleyensis*	+	+
				Orthocladius	*Orthocladius yagashimaensis*	+	+
					Orthocladius saxicola	+	+
					Orthocladius clarki	+	+
					Orthocladius sp.	+	
				Rheocricotopus	*Rheocricotopus* sp.	+	+
				Parametriore	*Parametriore* sp.	+	
				Psectrocladius	*Psectrocladius sp.*		+
	Malacostraca	***Amphipoda***	***Gammaridae***	*Anisogammarus*	*Anisogammarus* sp.	+	+
	Arachnida	***Acariformes***	***Hydrachinidia***	*Hydracarina*	*Hydracarina* sp.	+	+
	**Gastropoda**	**Basommatophora**	**Planorbidae**	*Hippeutis*	*Hippeutis umbilicalis*	+	
**Mollusca**			**Lymnaeidae**	*Galba*	*Galba pervia*	+	+
	**Lamellibranchia**	**Veneroida**	**Corbiculidae**	*corbicula*	*Corbicula nitens*	+	+

Note: An asterisk "+" indicates the presence of the species in the current survey, while the absence of an asterisk signifies that the species was not detected during the present investigation.

Table S3. Redundancy analysis (RDA) results predict macroinvertebrate species composition using selected environmental variables. Abbreviations: "ASL" represents altitude; "W" denotes stream width; "WD" stands for water depth; "WFR" indicates water volume flow rate; "SDD" is the abbreviation for Secchi disk depth; "WT" represents water temperature; "pH" signifies pondus hydrogenii; "DO" stands for dissolved oxygen; "Cond" denotes conductivity; "ORP" is the abbreviation for oxidation-reduction potential; "TN" represents Total Nitrogen; "TP" stands for Total Phosphorus; "NH_3-N" indicates Ammonia Nitrogen; "NO_3-" represents Nitrate Ion; and "COD" is the acronym for Chemical Oxygen Demand.

**Factors**	**Dry Season**				**Rainy Season**			
	**RDA1**	**RDA2**	**R^2**	**Pr(>r)**	**RDA1**	**RDA2**	**R^2**	**Pr(>r)**
**ASL**	0.90491	0.4256	0.4176	0.001	0.77386	0.63336	0.5322	0.001
**W**	-0.64275	0.76607	0.3138	0.002	0.84393	-0.53645	0.3762	0.001
**WD**	-0.94534	0.32609	0.2527	0.007	0.49802	-0.86717	0.3291	0.002
**SDD**	-0.77662	-0.62997	0.1183	0.059	-0.70122	0.71294	0.2831	0.008
**WFR**	-0.86114	-0.50837	0.2228	0.009	-0.85664	-0.51592	0.3745	0.001
**WT**	0.95757	0.2882	0.0509	0.406	0.66341	-0.74825	0.4609	0.001
**DO**	-0.83777	-0.54602	0.3772	0.001	-0.60842	-0.79361	0.0983	0.178
**Cond**	0.09285	0.99568	0.1096	0.118	0.68188	-0.73146	0.1874	0.032
**pH**	0.86303	0.50516	0.1895	0.015	0.95181	0.30668	0.1086	0.121
**ORP**	0.5225	0.85264	0.0795	0.221	0.18868	0.98204	0.1573	0.067
**TN**	0.05578	0.99844	0.0439	0.442	-0.47242	0.88137	0.0044	0.935
**TP**	-0.99556	-0.09411	0.0161	0.742	-0.82556	-0.56431	0.0904	0.172
**NH_3^-**	0.1075	0.99421	0.0733	0.285	-0.90459	-0.42628	0.0467	0.452
**NO_3-N**	0.09297	0.99567	0.1744	0.036	-0.43431	0.90076	0.0671	0.307
**COD**	-0.14112	0.98999	0.0789	0.226	-0.18466	0.9828	0.0187	0.679
